# A novel mutation in exon 2 of *FGB* caused by c.221G>T^*†*^ substitution, predicting the replacement of the native Arginine at position 74 with a Leucine (p.Arg74Leu^*†*^) in a proband from a Kurdish family with dysfibrinogenaemia and familial venous and arterial thrombosis

**DOI:** 10.1007/s11239-016-1439-z

**Published:** 2016-11-03

**Authors:** Abdul A. Shlebak, Alexia D. Katsarou, George Adams, Fiona Fernando

**Affiliations:** Haemostasis and Thrombosis Unit, Department of Haematology, Imperial College Healthcare NHS Trust Hospitals, St Mary’s Hospital Campus, Praed St, London, W2 1NY UK

**Keywords:** Dysfibrinogenaemia, Fibrinogen, Thrombosis, Novel mutation

## Abstract

Dysfibrinogenaemias may present in either congenital or acquired form and are disorders of fibrinogen structure which may or may not be associated with abnormal function. More than 100 point mutations with single amino acid substitutions have been identified in over 400 families. These lead to defective DNA in the translated fibrinogen molecule. Such cases have improved our understanding of the fibrinogen–fibrin structure. Six members of a consanguineous family including a female proband, a female sibling, three male siblings and a daughter, with ages between 29 years and 53 years presented with early onset venous and premature arterial thromboembolic disease were investigated for a pro-thrombotic tendency associated with dysfibrinogenaemia. The family was investigated using standard coagulation assays and DNA sequencing of the genes encoding the *FGA, FGB* and *FGG*. All cases have dysfibrinogenaemia with a fibrinogen level 1.4 to 1.5 (1.9–4.3 g/L). Thrombophilia testing (including AT, PS & PC, *F5 G1691A* (FV Leiden)/*F2* (prothombin G20210A) genotypes, homocysteine, antiphosphlipid antibody, paroxysmal nocturnal haemoglobinuria by flow cytometry and Janus Kinase-2 (exon 14)) were normal. PCR amplification and sequencing of exon 2 of *FBG* revealed a heterozygous mutation for a c.221G> T^***†***^substitution, predicting the replacement of the native Arginine at position 74 with a Leucine (p.Arg74Leu^***†***^). In silico analysis of p.Arg74Leu strongly support pathogenicity. A novel mutation was identified in exon 2 of *FGB* caused by c.221G> T^***†***^ substitution, predicting the replacement of Arginine at position 74 with a Leucine (p.Arg74Leu^***†***^) in a proband from a Kurdish family with dysfibrinogenaemia and familial venous and arterial thrombosis.

## Introduction

The generation of thrombin is the catalyst for the final step in the coagulation cascade for the conversion of soluble fibrinogen into insoluble fibrin polymer by the cleavage of the amino-termini of the α and β chains of fibrinogen, releasing fibrinopeptide A and B. The cleavage of fibrinopeptide A exposes new sites on the complex large fibrinogen molecule (composed of two identical subunits linked by a disulfide bond), these sites have high affinity for other fibrin molecules, leading to spontaneous fibrin polymerization with the formation of protofibrils. Fibrinopeptide B is released simultaneously with fibrinopeptide A but is not required for fibrin polymerization. The growing polymer is consolidated by the covalent bonding of the fibrin strands together through the action of the thrombin activated-transglutaminase factor XIIIa leading to stabilization of the friable platelet plug on which the fibrin polymer is deposited, later this is lysed by the fibrinolytic system [1].

Given that fibrinogen plays a pivotal role in both the pro-coagulant and fibrinolytic pathways, defects in fibrinogen function may be associated with increased risk for both haemorrhage and thrombosis. Congenital dysfibrinogenaemias, is a qualitative congenital fibrinogen disorder characterized by normal antigen levels of a dysfunctional fibrinogena [2]. It is a disorder of fibrinogen structure which may or may not be associated with abnormal function. Over 400 families with the congenital form [3] have been studied in whom structural defects were determined in approximately half of the cases [3–5]. The defects mostly result from DNA point mutations with single amino acid substitutions. Clinically, approximately half of affected individuals are asymptomatic [3, 4, 6] with a bleeding tendency in a quarter and the remaining patients experience thrombosis with or without haemorrhage. In one study 55 % (n = 250) of patients were found incidentally [4]. Similarly, in a UK report 57 % (n = 35) were asymptomatic [7], and in the recent long-term study of 101 patients 58 % had an incidental diagnosis, either during routine laboratory testing or before surgery [8]. The frequency of thrombosis was found to be higher in young women (mean age of first thrombosis was 27 years) with a high incidence of pregnancy-related thrombosis, especially in the postpartum period. Lower limb deep venous thrombosis predominates. In addition to an increased risk for thrombosis, there is a high frequency of pregnancy loss due to spontaneous abortions [9].

Although early studies involving over 2300 patients were insufficient to confirm a cause-effect relationship between dysfibrinogenaemia (reported in 1 % of patients) and VTE [6], later the International Society on Thrombosis and Haemostasis Scientific and Standardization Committee Subcommittee on Fibrinogen study convincingly supported causative association [6]. In a study of dysfibrinogenaemia with thrombophilia, 26 cases were evaluated along with 187 relatives (99 had dysfibrinogenaemia and 88 did not), in which 20 of the relatives with dysfibrinogenaemia experienced a thrombotic event compared with none of those without dysfibrinogenemia. Additionally, several cases had >2 family members with both dysfibrinogenemia and thrombosis at a young age.

Investigations for congenital dysfibrinogenaemia include the thrombin time which is abnormally prolonged in all but few exceptions, this is considered the most sensitive screening test [3, 10]. In one study of 35 probands with dysfibrinogenemia from 15 families, the prothrombin time and the activated partial prothrombin time were found to have lower sensitivities of 96 and 65 %, respectively [11]. The prolonged thrombin time however have low specificity as it is prolonged for a variety of reasons [12] including; the presence of heparin [13] fibrin degradation products, excess fibrinogen, paraprotein [14] acquired antibodies to bovine thrombin, [15] excess protamine, autoantibodies against fibrinogen functional domains [16, 17] primary amyloidosis [18] warfarin therapy [19] and hypoalbuminemia (<20 g/L) [20]. Reptilase time which uses an enzyme from snake venom (*Bothrops atrox*) instead of thrombin which cleaves fibrinopeptide A generating a clot. The reptilase time is used to detect heparin effect [21] where the reptilase time is consistently normal. Fibrinogen may be measured by functional (clot-based) method measuring only the functional fibrinogen that participates in clot formation, this is the preferred assay when evaluating dysfibrinogen [22]. Other methods include immunologic (antigenic) or chemical (precipitation). A truly low fibrinogen level may be seen in consumptive states, dilutional coagulopathy, fibrinolytic therapy, severe hepatocellular disease, hereditary hypofibrinogenemia or afibrinogenemia, and dysfibrinogenemia.

Genetically, the three fibrinogen polypeptide chains (Aα, Bβ, and γ) are encoded by 3 separate genes on the long arm of chromosome 4 with autosomal dominance mode of transmission of the various hereditary dysfibrinogens except in rare conditions [23]. In addition to its role in haemostasis, fibrinogen is also involved in wound healing, inflammation, cell migration and proliferation. Fibrinogen mutations have been implicated in the pathogenesis of other unrelated conditions including in hereditary amyloidosis [3]. Further information on fibrinogen mutation can be obtained from this link (http://www.geht.org/databaseang/fibrinogen) [24].

## Case report

**Fig. 1:**
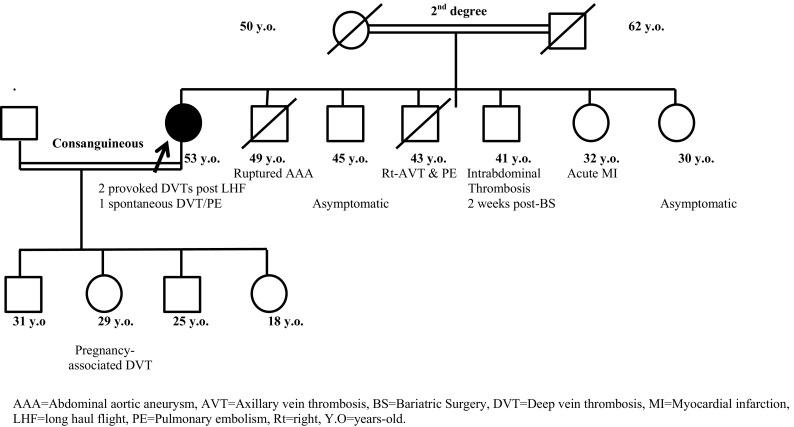
Family pedigree

A 53 year-old female (Proband (case#1)) presented with first episode of deep vein thrombosis (DVT) two days following a 9-hour long haul flight at the age of 41 year, a first recurrence occurred 3 years later following the same trigger. These two episodes were managed with a limited period of anticoagulation. A further spontaneous recurrence of DVT and PE (first spontaneous event) was later diagnosed and is now established on long-term anticoagulation with the vitamin-K antagonist warfarin. The family pedigree is shown in Fig. 1. The clinical characteristics of the other five affected members of the family are shown in Table 1. The table describes the thrombotic episode(s) in relation to site, type and the presence of triggering event and other risk factors. Case#6 sustained a VTE episode despite appropriate thromboprphylaxis with enoxaparin 0.6 mg/kg daily S/C perioperatively for bariatric surgery. Figure 1 and Table 1 summarises the relevant clinical characteristics and the family pedigree.

**Table 1:** Clinical characteristics of affected family members

	Case#1	Case#2	Case#3	Case#4	Case#5	Case#6
Age (years)	53	49	43	41	31	29
Gender	F	M	M	M	F	F
Relation to proband	Proband	Sibling	Sibling	Sibling	Sibling	Daughter
History of venous thrombosis	Yes	No	Yes	Yes	No	Yes
History of arterial thrombosis	No	Yes	No	No	Yes	No
Site of thrombosis	Leg DVT and PE	Rupture of AAA	Axillary vein thrombosis and PE	PE	Acute Coronary syndrome	Leg DVT
Provoking trigger	Yes in 2 episodes-long haul flight	No	No	Yes	No	Yes
Spontaneous event	Yes-one	Yes	Yes	No	Yes	No
Timing of thrombosis	2 days post long-haul flight	n/a	n/a	14 days post-surgery	n/a	8 weeks gestation
Other risk factors	Yes	No	Smoker	Smoker Surgery Obesity	No	Pregnancy
Outcome	On life-long anticoagulation Post-thrombotic syndrome	Fatal	Fatal	On anticoagulation To assess duration	On dual antiplatelet therapy	Under surveillance

*AAA* abdominal aortic aneurysm, *DVT* deep venous thrombosis, *F* female, *M* male, *n*/*a* not applicable, *PE* pulmonary embolism

## Methods and results

### Coagulation screen and d-dimer

In our Trust routine coagulation screen consists of a Prothrombin Time (PT), Activated Partial Thromboplastin Time (APTT) and a Clauss Fibrinogen (FIB) assay [25]. Thrombin Time (TT) and Reptilase time (RT) are also performed upon request. Venous blood samples were collected into 0·105 M tri-sodium citrate containing bottles and analysed using Sysmex coagulometers (Sysmex Corporation, Hamburg, Germany; CS2100i) with Dade-Behring (Marberg, Germany) reagents (PT—Innovin; APTT—Actin FSL; TT—Thromboclotin reagent (1·25 μ/ml bovine thrombin); FIB—Thrombin reagent (10 IU/ml).

The fully automated, computerised blood coagulation Sysmex analyser CS2100i uses closed vial sampling, utilises a photo-optical detection method by passing multiple wavelengths of light delivered by fibre-optic cable through the patient-reagent mixture. The analyser detects changes in transmitted light intensity as the end-point conversion of fibrinogen to fibrin results in an increase in optical density. The coagulation curve is drawn using time as the X axis and transmitted light intensity as the Y axis. The clotting time end-point (in seconds) is determined where a 50 percent change in optical density (OD) is reached for the PT, APTT, TT (660 nm) and FIB (405 nm).

The Innovance® immunoturbidimetric D-dimer assay employs polystyrene particles coated with D-dimer specific monoclonal antibodies. When mixed with the test plasma, an antigen–antibody reaction takes place, leading to agglutination of the latex microparticles in the presence of D-dimers resulting in an increase in turbidity which is detected as an increase in optical density (OD) measured at 800 nm. The increase in OD is proportional to the level of D-dimer in the test sample where the delta change in OD is compared against a standard curve to quantitate D-dimer levels.

### Thrombophilia tests

Venous blood samples were collected with minimal stasis using a 19-gauge butterfly needle into 0.105 M trisodium citrate. Platelet poor plasma was prepared by double centrifugation of samples at 2700×g at room temperature for 20 min. The plasma is then separated into freezer tubes aliquots (1.5 ml) and stored at −70 °C.

We determined antithrombin and protein C activities, free protein S antigen, *F5* (FV Leiden) G1691/*F2* (prothrombin gene) G20210A genotypes and antiphospholipid screen comprising lupus anticoagulant, anticardiolipin and anti-β2GP-1 antibody in three subjects with objectively documented thrombosis. Table 2 summarises blood results for all general and prothrombotic work-up.

**Table 2:** Summary of blood results

Test	Normal range	Index Case#1	Case#4	Case#6
White blood cell count	4.2–11.2 × 10^9^/L	5.9	7.7	8.3
Haemoglobin	114–150 g/L	126	148	125
Platelet count	135–400 × 10^9^/L	226	233	234
Prothrombin time (PT)	9.4–11.3 s	11.9	12.1	13.0
Activated partial thromboplastin time (APTT)	25.0–30.7 s	28.1	32.1	25.4
Thrombin time (TT)	12.9–15.2 s	26.2	25.7	23.4
Reptilase time (RT)	15–19 s	27.1	28.3	26.7
Fibrinogen	1.9–4.3 g/L	1.51	1.62	1.55
D-dimer	<560 ug/L	238 ug/L	217	169
Anti-thrombin activity	0.80–1.4 iu/L	0.84	1.2	0.97
Protein-C activity	0.65–1.35 iu/L	1.32	1.17	1.28
Protein-S-free antigen	0.66–1.54 iu/L	0.79	0.94	0.88
Factor V Leiden genotype	G/G	G/G	G/G	G/G
G20210A	G/G	G/G	G/G	G/G
Factor VIII activity	0.6–1.43 iu/L	0.71	1.30	1.17
Fibrin plate lysis	0.65–1.20	1.29	n/a	n/a
Homocysteine	5–15 umol/L	12.0	10.7	8.3
Antiphospholipid antibody Screen	Lupus Anticoagulant (dRVVT) Anticardiolipin antibody Anti-β2GP-I	Negative Negative Negative	Negative Negative Negative	Negative Negative Negative
Activated protein C resistance	2.10–4.60	3.36	4.0	2.73
Modified activated protein C resistance	2.20–2.70	2.91	2.5	2.1
PNH immunophenotyping	Granulocyte CD24 and FLAER Monocyte CD14 and FLAER Red cells CD 59	No clone detected	No clone detected	No clone detected
JAK-2 exon-14	5 % detection level by PCR	Not detected	Not detected	Not detected
Fibrinogen gene mutation analysis		Exon 2 of FGB caused by c.221G> T substitution	Not done	Not done

*Anti-β2GP-I* anti-β 2Glycoprotein-I, *FLAER* fluorescent aerolysin, *dRVVT* dilute Russell Viper Venom Test, *n*/*a* not available

### Chromogenic protein C and antithrombin assays

As described earlier, the analyser detects changes in transmitted light intensity at 405 nm. The optical density increases due to the increase in colour change caused by the cleaving of the respective chromogenic substrate (for protein C or AT) releasing pNA (p-nitroanilide). The increase in colour change is proportional to the level of Protein C or Antithrombin activity present in the respective sample.

### Free protein S antigen assay

Monoclonal antibodies specific for free protein S are incorporated using Innovance® immunoturbidimetric method to quantitate levels of free Protein S as described above under D-dimer quantitation.

### Activated protein C resistance (APCr)

APC resistance was assessed by measuring the anti-coagulant response in plasma on the addition of APC (Dade ProC Global Kit). A ratio of <2.10 for the clotting time in the presence of APC/clotting time in the absence of APC was taken to represent APC resistance as described by Rosen et al [26]. Modified APCr (<2.20) is obtained as above but using Factor V deficient plasma, this is very sensitive to factor V mutations including Factor *V* Leiden.

### Factor V Leiden and prothrombin G20210A

For factor V Leiden, exon 10 of the factor V gene was amplified by PCR using known primers, which contains the mutation G→A at nucleotide position 1691 as described by Bertina et al., 1994 [27]. The amplified DNA segment was then digested using the restriction enzyme MnlI (New England BioLabs) overnight at 37 °C, followed by electrophoresis separation in a 1.8 % agarose gel stained with ethidium bromide at a potential difference of 100 V and then examined under UV light. PCR amplification using specific primers generates a 267-bp fragment spanning the mutation site. The G1691A mutation on the factor V gene destroys a MnlI cleavage site. Digestion of the amplified fragment with MnlI generates three fragments—37, 67 and 163 bp in length—in the presence of normal factor V genotype. In the presence of the affected allele, digestion with MnlI generates two fragments—67 and 200 bp in length. A heterozygous sample would therefore generate four bands—37, 67, 163 and 200 bp in length—and a homozygous sample would generate only two bands—67 and 200 bp in length.

For prothrombin G20210A, PCR using specific primers was used to amplify a 345-bp segment, followed by overnight digestion with the restriction endonuclease HindIII at 37 °C, followed by gel electrophoresis (Poort et al., 1996) [28]. The G20210A mutation in the 3′ untranslated region of the prothrombin gene does not disrupt a natural recognition site for any restriction endonuclease. The primers used are designed to introduce a HindIII cleavage site only if the mutant allele is present. Following digestion with HindIII one fragment of 345 bp is yielded in a normal individual, two fragments of 322 and 23 bp in a homozygous individual and three fragments of 345, 322 and 23 bp in a heterozygous individual.

### Antiphospholipid antibodies

Lupus anticoagulant is detected using the dilute Russell’s viper venom time (dRVVT) together with a neutralization step with phospholipid. Patient samples with a dRVVT ratio (test/control) of ≥ 1.2 were retested with a neutralization step with phospholipid. A decrease of 10 % or more in the ratio was considered to be positive for lupus anticoagulant [29]. Anticardiolipin and anti-β2GP-1 antibodies and were identified using a standardized enzyme linked immunosorbent assay (ELISA) [30, 31]. Initial positive test for LA, positive aCL titre, or positive anti-β2GP-1 antibody had a confirmatory test performed on a second sample taken at least 12 weeks later. Only patients with persistently positive tests were considered to have the antiphospholipid syndrome.

### Analysis of the fibrinogen genes

Causative single nucleotide variations (SNVs) were sought by polymerase chain reaction amplification and sequencing of FGA exon 2, FGG exon 2 and FGG exon 8, which are hotspots in the GEHT dysfibrinogenaemia mutation database [24]. The remaining coding sequences of FGA, FGB and FGG were sequenced in subjects with previously unreported candidate SNVs. In our family, PCR amplification and sequencing of exon 2 of *FBG* showed the proband to be heterozygous for a c.221G> T^*†*^ substitution, predicting the replacement of the native Arginine at position 74 with a Leucine (p.Arg74Leu^*†*^) (LSDB:http://www.geht.org/fr/pages/set_pratique03a.html). This is a novel mutation. Analysis of the coding regions of *FGA, FGB, FGG* has been completed and no additional putative mutations identified.

### In silico analysis

In silico is an expression used to indicate the of computer software simulation to predict impact of an abnormaility inclusing genetic mutations. The expression in silico was first used in 1989. This is now widely used by diagnostics laboratories as a splice prediction tools to predict the effect of a genetic variant. In our study, in silico analysis using ALAMUT software (http://www.interactive-biosoftware.com/doc/alamut-visual/2.8) predicted that p.Arg74Leu mutation strongly support pathogenicity [32].

## Discussion

The exact mechanism by which dysfibrinogenemia increases thrombosis risk is not well understood, biologically, this may occur due to either excessive thrombosis through increased thrombin generation and clot formation or impaired fibrinolysis and clot lysis. Mechanistically this depends on the specific fibrinogen defect. Defective binding of thrombin by the abnormal fibrinogen molecule was postulated resulting in excess circulating thrombin which activates platelet [33–35]. Alternatively, impaired fibrinolysis as a result of defective binding of tissue-type plasminogen activator [35] or lysis-resistance to plasmin [36, 37] has been suggested. The prothrombotic correlation of dysfibrinogens is found in the following fibrinogens including; Caracas V, Chapel Hill III, Christchurch II & III, V Marburg, Germany-k, Hannover II, Ijmuiden, Melun, Milano III, New York I, Nigmegen, London VII, Paris V, Lissingen/Frankfurt IV [3]. These 15 “thrombophilic” dysfibrinogens have mutations that are predominantly found in the C-terminal domain of the Aα chain and the thrombin cleavage site of the Bβ chain [6].

Molecular defects due to heterozygous missense mutations localized in exon 2 of FGA and exon 8 of FGG, lead to defects in fibrinogen to fibrin conversion, fibrin network formation, and other fibrinogen functions. As most individuals with the condition are heterozygous, they have circulating mixture of normal and abnormal fibrinogen. Moreover, the molecular basis for the defect and the resulting symptoms (genotype-phenotype correlation) vary greatly. The analysis of dysfibrinogens at the molecular level has been challenging due to the size and complexity of the fibrinogen molecule but overall the defects can be separated into two major groups. Those which affects the release of the fibrinopeptides A and B, and those that do not. The first group tends to associate with bleeding complications because they interfere with the initial conversion of soluble fibrinogen to fibrin monomer [2] and they account for the majority of abnormal fibrinogens and include substitutions of amino acids situated at the amino-terminal regions of the α and β chains, specifically at or near the thrombin-cleavage sites [38]. The other more heterogeneous group, vary clinically from asymptomatic, to severe bleeding, to severe thrombophilia and comprises mutations within the globular carboxyl-terminal regions of the three chains as well as mutations at the polymerization sites. The genotype-phenotype correlation in this group have not been well characterized.

It is now accepted that the symptoms associated with various dysfibrinogenemias may be modulated by coexisting factors. As predisposition to thrombosis is often multifactorial and may be the result of more than one mutation in genes encoding haemostasis proteins [39], thrombophiliac dysfibrinogenemia may interact synergistically with other genetic and non-genetic risk factors such as trauma, pregnancy, oral contraceptives, hormone replacement, surgery and cancer. Several examples illustrate this phenomenon including the heterozygous dysfibrinogemias Cedar Rapids (R275C) [40] and Giessen IV (D318G) [5] both of which were reported in association with a heterozygous factor V Leiden mutation. In the Cedar Rapids family, either defect alone was not associated with symptoms, but the double mutation was strongly associated with pregnancy-related thrombosis. The complex interactions with potential risk factors would explain partially the variability observed in the clinical symptoms. In the case of the D364, N308 and R275 mutations, the clinical manifestations associated with a given molecular defect varied greatly from one patient to another from being silent or with mild haemorrhage to severe thrombophilia [41]. As few genotypes are clearly correlated with a clinical phenotype [5], this makes the clinical management a great challenge. Even asymptomatic persons are at risk of developing cardiovascular and/or major bleeding events during the natural course of the disease [8].

To our knowledge this novel is a unique mutation in this ethnic population. The Kurdish population is known for its common practice of consanguinity, therefore, this is likely to be a prevalent finding. In our family, thrombosis affected both genders in keeping with autosomal dominance pattern with both venous and arterial thrombosis presenting prematurely with age ranging from 29 to 49 years. In common with other similar conditions, there is a demonstrable trigger for at least four of venous thrombotic episodes varying from Long-haul travel to pregnancy-associated. One male sibling sustained a post-operative PE following bariatric surgery despite carful perioperative thromprophylactic strategy. Two male deaths occurred at 43 and 49 years due to fatal pulmonary embolism and ruptured atheomatous abdominal aortic aneurysm respectively. In our patients, the preliminary coagulation screens including significantly prolonged thrombin time and low functional fibrinogen concentration were highly suggestive of dysfibrinogenaemia. As in this family, thrombophilic defect are equally important to consider in view of young age and the disproportionate degree of the thrombotic episodes in relation to the provoking events. This is a novel thrombophilic fibrinogen which is found in a defined ethnic group of Kurdish descent and the finding of low fibrinogen in individuals of such ethnic group would benefit them from the appropriate thrombosis risk analysis and advice for risk reduction measures for both venous and arterial thrombosis including the avoidance of estrogen contraceptives and implementing aggressive thromboprophylaxis. Our paper is significantly limited by the lack of genotype information on other members of the family, affected or unaffected, the lack of motivation in other members of the family in providing blood samples coupled by concerns about labeling them with a potential genetic condition presented an obstacle for this despite clinical counseling. Furthermore, the cost of the analysis is not covered under routine national health service (NHS) pathology funding. The fibrinogen gene analysis is performed in a regional genetic laboratory hosted by another institute, in contrast to the thrombophilia testing which is performed in house. Further investigations with other patients from similar ethnic background will hopefully shed more light on the significance of this genetic mutation.

## Conclusions

It is critical to evaluate the contribution of other risk factors in patients with dysfibrinogenemia. This is to ascertain if the dysfibrinogenemia is the sole thrombotic risk factor present in each individual. The elucidation of crystal structures of fibrinogen fragments has shed further light on the arrangement of domains and the interactions between amino acid residues that are vital for the various functions of this complex molecule. The effects of mutations that cause dysfibrinogenemias can now be explored through further structure–function studies, functional analysis of polymerization and fibrinolysis, viscoelastic properties of fibrin clot. Future research will lead to a better understanding, diagnosis and management of the condition.
